# Mechanism of potassium ion uptake by the Na^+^/K^+^-ATPase

**DOI:** 10.1038/ncomms8622

**Published:** 2015-07-24

**Authors:** Juan P. Castillo, Huan Rui, Daniel Basilio, Avisek Das, Benoît Roux, Ramon Latorre, Francisco Bezanilla, Miguel Holmgren

**Affiliations:** 1 Laboratorio de Fisiología Celular, Facultad de Ciencias, Universidad de Chile, Montemar 254006, Chile; 2 Centro Interdisciplinario de Neurociencia de Valparaíso, Universidad de Valparaíso, Valparaíso 2366103, Chile; 3 Department of Biochemistry and Molecular Biology, University of Chicago, Gordon Center for Integrative Sciences, Chicago, Illinois 60637, USA; 4 Facultad de Ciencias, Universidad de Chile, Santiago 7800003, Chile; 5 Molecular Neurophysiology Section, Porter Neuroscience Research Center, National Institute of Neurological Disorders and Stroke, National Institutes of Health, Bethesda, Maryland 20892, USA

## Abstract

The Na^+^/K^+^-ATPase restores sodium (Na^+^) and potassium (K^+^) electrochemical gradients dissipated by action potentials and ion-coupled transport processes. As ions are transported, they become transiently trapped between intracellular and extracellular gates. Once the external gate opens, three Na^+^ ions are released, followed by the binding and occlusion of two K^+^ ions. While the mechanisms of Na^+^ release have been well characterized by the study of transient Na^+^ currents, smaller and faster transient currents mediated by external K^+^ have been more difficult to study. Here we show that external K^+^ ions travelling to their binding sites sense only a small fraction of the electric field as they rapidly and simultaneously become occluded. Consistent with these results, molecular dynamics simulations of a pump model show a wide water-filled access channel connecting the binding site to the external solution. These results suggest a mechanism of K^+^ gating different from that of Na^+^ occlusion.

## Abstract (web summary)

During transport by the Na^+^/K^+^-ATPase, Na^+^ and K^+^ ions become occluded between intra- and extracellular gates. Here Castillo *et al.* measure transient electrical signals arising from K^+^ occlusion and use molecular simulations to describe a K^+^ gating mechanism fundamentally different to that of Na^+^.

## Graphical abstract

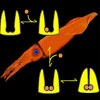

Crystal structures of the Na^+^/K^+^-ATPase show that ions are bound and occluded deep within the protein about 60% of the way through the pore from the extracellular medium^1^,^2^,^3^,^4^. Ions reach their binding sites through hydrophilic paths called access channels. Most of the Na^+^/K^+^-ATPase’s voltage dependence originates as ions move along these access channels sensing the electric field across the membrane. The kinetic, thermodynamic and electrical properties of these access channels and their associated occlusion/deocclusion transitions for Na^+^ ions have been well characterized by both electrophysiological and spectroscopic approaches^5^,^6^,^7^,^8^,^9^,^10^,^11^,^12^,^13^,^14^,^15^,^16^. These Na^+^-dependent signals are large and relatively slow. Transient electrical signals mediated by the binding/release of external Na^+^ have been particularly useful in dissecting multiple occlusion/deocclusion events as Na^+^ ions are released to the extracellular medium^8^,^9^,^11^. The electrogenicity of K^+^ binding has also been established^5^,^14^,^16^,^17^,^18^,^19^,^20^. Nonetheless, direct measurements of transient electrical signals mediated by K^+^ ions have been difficult to detect^13^,^21^, presumably because these transient electrical signals are small and fast^20^. There are no available electrophysiological recordings of K^+^-mediated currents associated with external K^+^ binding/occlusion. However, to slow down the kinetics of these transitions, Peluffo and Berlin^20^ substituted external K^+^ by Tl^+^, a congener K^+^ ion capable of being transported by the Na^+^/K^+^-ATPase^22^. The properties of these transient currents were consistent with ions traversing the electric field through access channels. Further support of external K^+^ ions moving through access channels comes from observations that quaternary ammonium ions, although able to compete with external K^+^ binding, they are not occluded, and that the binding and unbinding kinetics were voltage dependent^18^,^19^.

Using high-speed voltage clamp and large squid giant axons (>1 mm diameter), we were able to characterize transient currents mediated by the binding and occlusion of external K^+^. Indeed, the electrical signals reported here are smaller (∼5 times) and much faster (∼10 times) than the corresponding transient currents carried by external Na^+^. The amount of charge moved and the kinetics of these K^+^-mediated transient currents are best described by an access channel model in which two K^+^ bind sequentially but they occlude simultaneously by a voltage insensitive step. Using molecular dynamic simulations, we dissect the electrical contributions of each K^+^ as they travel through the access channel to their binding sites, providing a consistent molecular picture of the functional data.

## Results

### Extracellular K^+^-mediated charge movement

**Figure 1:**
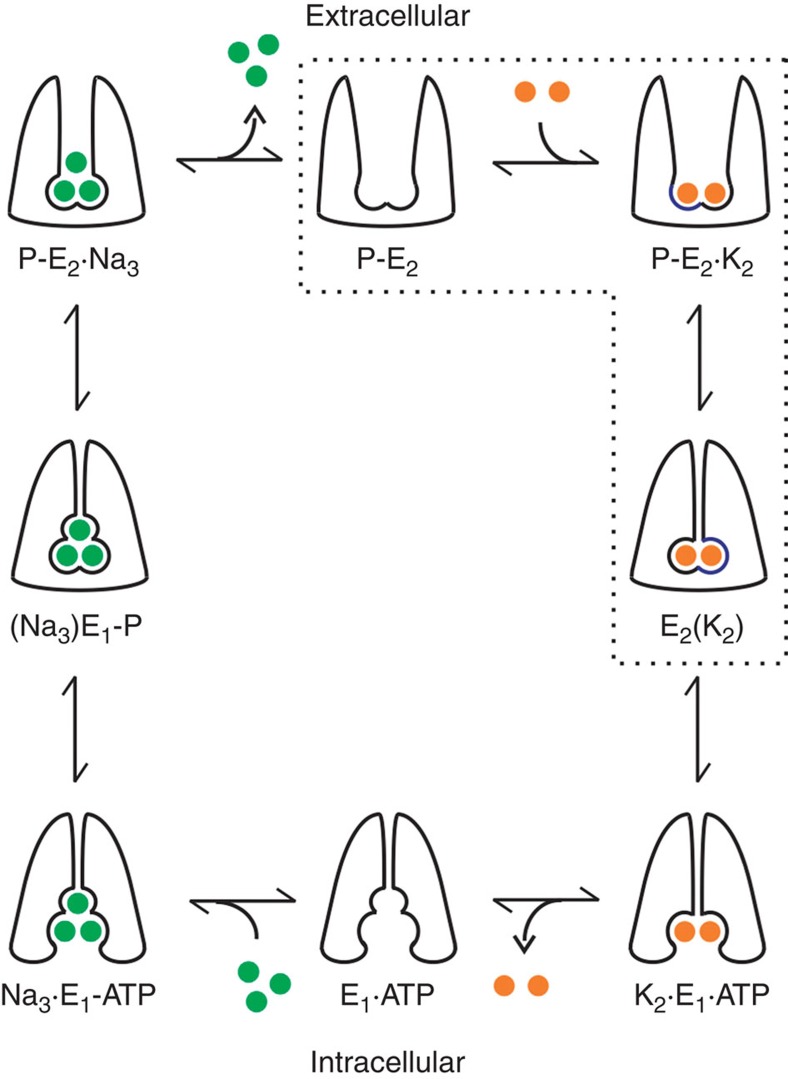
Albers–Post model for the Na^+^/K^+^pump transport mechanism. Three Na^+^ (green circles) moved out followed by two K^+^ transported in, through a series of states that alternate the binding sites at both sides of the cell membrane, which are associated with phosphorylation and dephosphorylation reactions. Experimental solutions were designed to populate states encircled by dotted lines. First, Na^+^ was removed from extracellular and intracellular solutions. Second, the intracellular solution contained 100 mM K^+^, 5 mM ATP, 15 mM Mg^2+^ and 5 mM P_i_.

**Figure 2:**
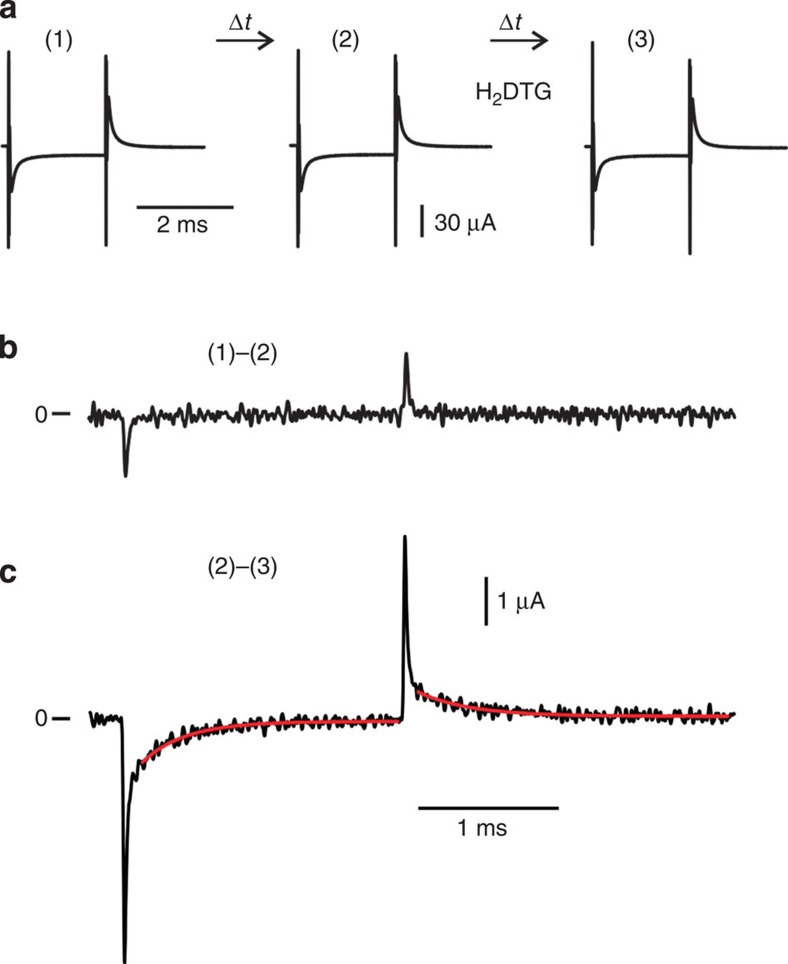
Charge movement mediated by extracellular K^+^. (**a**) Experimental protocol. Current traces (1)–(3) represent the total membrane current in response to a voltage step from 0 to −160 mV acquired at 2 μs per point, filtered at 100 kHz, in the presence of 1 mM external K^+^ and at equal time intervals. Between traces (2) and (3), 100 μM H_2_DTG was applied to the external solution. (**b**) Pump-mediated K^+^ charge movement: time control. Subtraction of trace (2) from (1) provides an evaluation for the stability of the preparation. Time control signals have temporal decays comparable to the voltage clamp speed and they likely originate from changes in series resistance^69^. Trace was digitally filtered at 25 kHz for display. (**c**) Pump-mediated K^+^ charge movement: H_2_DTG-sensitive transient currents. Current trace corresponds to the charge movement during binding/occlusion of external K^+^. Solid red lines represent single exponential fits with time constants of 306 and 370 μs at −160 and 0 mV, respectively. Trace was digitally filtered at 25 kHz for display.

**Figure 3:**
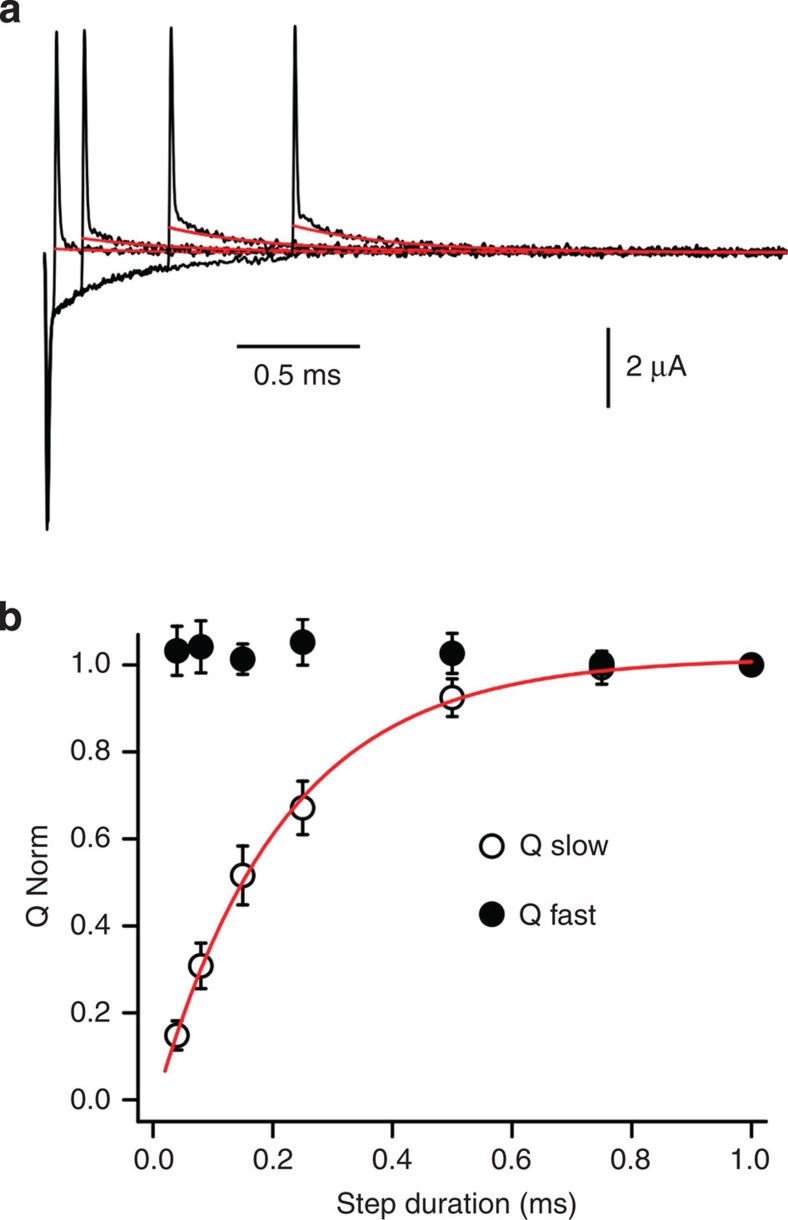
Fast and slow components of K^+^ charge movement are kinetically independent. (**a**) Experimental protocol. Superimposed H_2_DTG-sensitive currents in response to a voltage jump to −160 mV with step durations of 40, 150, 500 and 1,000 μs. Solid red lines represent monoexponential fits (*τ*=408 μs) of the current decays on return to 0 mV after each step. The time constant of the 1-ms pulse to −160 mV was 286 μs. (**b**) Normalized charge quantities moved by the slow and fast components measured at 0 mV. Charge moved by the slow component (*Q*_s_) increases monotonically with the duration of the step to −160 mV. The solid red line represents a monoexponential fit with *τ*_s_=212±9 μs. The fast component (*Q*_f_) remains essentially constant. The presence and magnitude of *Q*_f_ are not affected by the monovalent cations used to substitute Na^+^ (that is, TMA or *N*-methyl D-glucamine), nor by changes in the concentrations of Ca^2+^ or Mg^2+^. Supplementary Fig. 2c shows a potential model and simulations that explain the time course and persistence of *Q*_f_. We noticed that for pulses longer than 1 ms there was a small (∼10–15%) reduction of OFF charge carried by the slow component. Neither the time constant nor the extent of this reduction is influenced by external K^+^, indicating that it is not related to external K^+^ binding and occlusion. *n*=4; scale bars represent s.d.

To study the properties of external K^+^ binding and the associated kinetics of K^+^ occlusion and deocclusion, we measured charge relaxation mediated by the Na^+^/K^+^-ATPase in squid giant axons from *Dosidicus gigas*. Axons were voltage clamped with time constants of ∼6 μs (Supplementary Fig. 1) and internally dialysed with solutions intended to restrict the Na^+^/K^+^-ATPase’s transport cycle to partial reactions involving extracellular K^+^ (Fig. 1). With 1 mM extracellular K^+^, Fig. 2a shows total membrane currents in response to a 2-ms voltage step to −160 mV from a holding potential of 0 mV, acquired at 500 kHz, filtered at 100 kHz and recorded at equal time intervals. Na^+^/K^+^ pump-mediated currents (Fig. 2c; (2)–(3) bottom) were extracted as the membrane current sensitive to dihydrodigitoxigenin (H_2_DTG), a specific and reversible squid Na^+^/K^+^ pump inhibitor^23^. On sudden changes in voltage to −160 mV and back to 0 mV, H_2_DTG-sensitive transient currents consist of two components (Fig. 2c): fast (comparable to the voltage speed of the clamp; Supplementary Fig. 2) and slow. The latter relaxed monoexponentially to near-zero current values (Fig. 2c; fits indicated by red solid lines) as expected by the ionic composition of the intracellular and extracellular solutions, which prevents completion of the transport cycle.

In contrast to Na^+^-occlusion process where the three charge translocation components’ quantities and time courses are tightly correlated^8^,^11^ (see also Supplementary Fig. 3), the fast component of K^+^-mediated charge movement appears to be unrelated to K^+^ transport. Figure 3a shows four superimposed H_2_DTG-sensitive transient currents in response to voltage steps from 0 to −160 mV of different durations before returning back to 0 mV. Irrespective of the length of the voltage step, the fast pump-mediated charge movement at the end of the pulse has similar magnitudes (Fig. 3b; filled circles), suggesting that the fast component of the charge movement does not represent a K^+^ binding/occlusion transition. A parsimonious explanation of this component is a change in the electric field produced by ouabain binding (Supplementary Fig. 2c). In contrast, the slow charge (*Q*_s_) increases monotonically with a time constant similar to the slow On relaxation (Fig. 3b; open circles). These results imply that the slow component of the H_2_DTG-sensitive transient currents (Figs 2c and 3a) should represent the binding/occlusion of two K^+^.

### Mechanism of extracellular K^+^ occlusion

**Figure 4:**
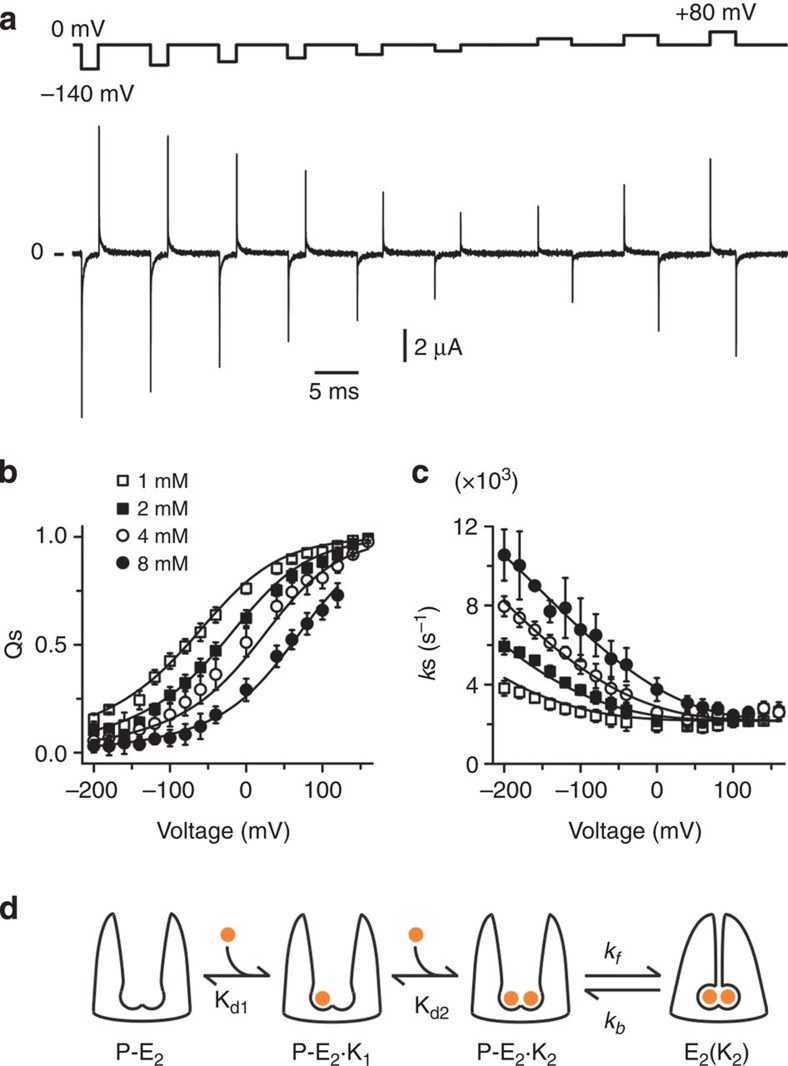
Voltage and external K^+^ dependence of the relaxation rate and charge moved by *Q*_s_ during K^+^ occlusion. (**a**) H_2_DTG-sensitive transient currents in response to a family of voltage steps from −140 mV to +80 mV in 1 mM external K^+^. (**b**) Charge displaced (*Q*_s_) versus voltage at different external K^+^ concentrations. (**c**) Relaxation rates (*k*_s_) versus voltage at different external K^+^ concentrations. (**b**,**c**) *n*=9, 12, 8 and 5 for 1, 2, 4 and 8 mM K^+^, respectively; scale bars represent s.d. (when not shown, s.d. was smaller than the symbol size). (**d**) Four-state access channel model with two consecutive K^+^-binding events that precede a single occlusion transition in which both ions are trapped within the permeation pathway of the pump. The two sequential voltage-dependent K^+^-binding reactions steps are fast and in equilibrium with the slow voltage-independent occlusion/deocclusion transition. In this model, the two K^+^-binding sites are assumed to be distinct with no cooperativity between them (see, for example, refs 16, 19). A global fit of this model to *k*_s_ and *Q*_s_ data (solid lines in b) gave the following best-fit parameter values with their 95% confidence interval in parenthesis: *K*_d1(0)_=2.6 (2.3, 3.0) mM, *K*_d2(0)_=42.7 (35.7, 49.7) mM, *λ*_1_=0.46 (0.43, 0.48), *λ*_2_=0.27 (0.25, 0.29), occlusion rate (*k*_f_)=13,450 (12,087, 14,813) s^−1^ and deocclusion rate (*k*_b_)=2,170 (2,095, 2,247) s^−1^; *r*^2^=0.99. In addition to have a more straightforward physical interpretation, this model fit the data statistically (F-test, *P*>0.99) better than the single binding/occlusion model of Supplementary Fig. 4.

Extending the range of membrane potentials (Fig. 4a), we studied the properties of the slow component at different external [K^+^]_o_. With the simple external K^+^ occlusion scheme enclosed by dotted line in Fig, negative voltages would drive K^+^ to their binding sites populating the E_2_(K_2_) state (Fig. 1). Conversely, positive potentials would release K^+^ from the enzyme driving the system in the direction of the P−E_2_ empty state and at extreme positive voltages no further recruitment of E_2_(K_2_) would be observed. Our results concur with these predictions (Fig. 4b). The velocity of the occlusion/deocclusion (*k*_s_) transition approaches a voltage-independent minimum of ∼2,000 s^−1^ at positive voltages and speeds up at extreme negative potentials approaching an extrapolated maximum of >12,000 s^−1^ (Fig. 4c). For intermediate voltages between the limiting cases, the relaxations become faster with increasing concentrations of external K^+^, reflecting the larger likelihood of K^+^ binding at negative potentials. The general features of these transient currents resemble the Na^+^ charge movement^9^,^10^,^11^,^24^,^25^,^26^, but with K^+^, instead of Na^+^. Thus, the data are consistent with the view that the movement of K^+^ through access channels between the external solution and the binding sites senses the voltage across the membrane, while the occlusion/deocclusion transition is essentially electroneutral (see also refs 18, 19, 20). In detail, however, there are substantial differences. Solid lines through the data (Fig. 4b,c) represent a simultaneous fit of charge quantities and relaxation rates of the access channel model shown in Fig. 4d (see Methods). The first difference is that the voltage-independent occlusion (*k*_f_∼13,000 s^−1^) and deocclusion (*k*_b_∼2,000 s^−1^) rates are notoriously faster for K^+^ than for Na^+^ translocation (*k*_occ_∼1,300 s^−1^ and *k*_deocc_∼100 s^−1^; see ref. 4). Faster rates for K^+^ occlusion would favour normal forward pumping in spite of external [K^+^] being about an order of magnitude lower than external [Na^+^]. Second, we have no evidence that the two K^+^ are occluded in distinct steps, as it occurs for Na^+^ translocation^8^,^11^. At all potentials and external [K^+^] tested, the slow component is properly fitted with one exponential component. These results indicate that the protein conformational change responsible for trapping (or occluding) both K^+^ in their binding sites occurs in a single step. There are two possible ways for two K^+^ ions travelling from the external bulk solution to their binding sites—they could either bind simultaneously, with unique fraction of the electrical field along the access channel (*λ*) and apparent dissociation constant at 0 mV (*K*_d(0)_); or they could bind in two separate steps in equilibrium with the occlusion/deocclusion transitions, each with its own *λ* and *K*_d(0)_. We have fitted our data to these two models (Fig. 4c; Supplementary Fig. 4). The best fit is achieved with a two-step binding/release model, as represented by solid lines in Fig. 4b. The best parameter fit values of the dissociation constant for the first and the second K^+^-binding site were 2.6 and 42.7 mM, respectively. These values are orders of magnitude lower than those estimated for Na^+^ when the pump exposes its binding sites to the extracellular milieu^11^,^24^,^26^, indicating that the two binding sites have a much higher affinity for extracellular K^+^ ions, which would support forward directionality of the transport process. The fraction of the electric field sensed by each K^+^ is 0.46 (*λ*_1_) and 0.27 (*λ*_2_) for the first and second K^+^ binding sites, respectively. This means that K^+^ ions sense a substantially smaller fraction of the electric field than the first Na^+^ released (*λ*∼0.7)^7^,^9^,^10^,^11^,^15^,^16^,^24^,^26^,^27^. Since crystal structures show that Na^+^ and K^+^ bind about the same distance from the external bulk solution^1^,^2^,^3^,^4^, these results may imply that the K^+^ ions travel along a wider external access channel.

### Structural model of the Na^+^/K^+^-pump access channel for K^+^

**Figure 5:**
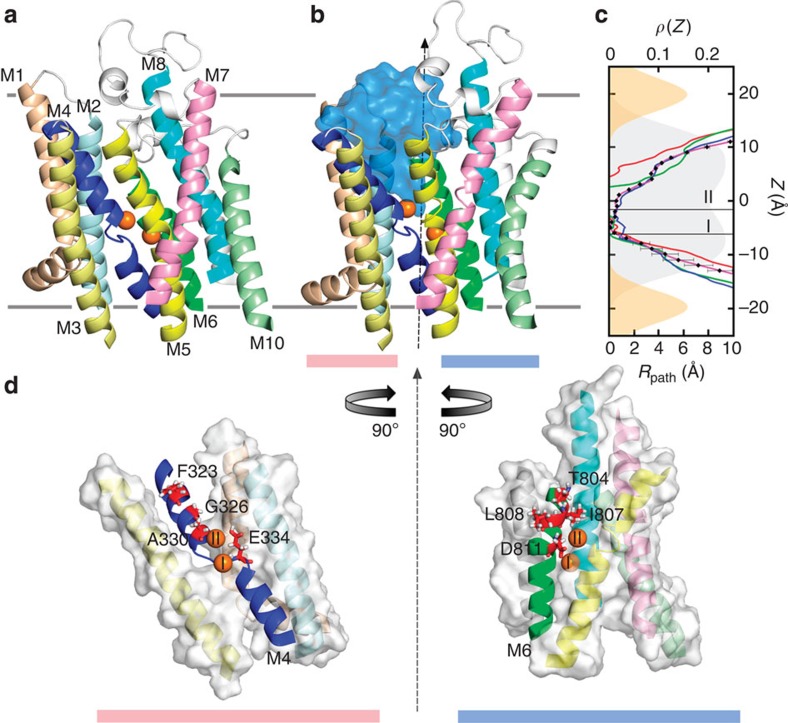
Extracellular occlusion–deocclusion of the Na^+^/K^+^-ATPase in the presence of bound K^+^. (**a**) Structure of the occluded E_2_(K_2_) state of the pump after 300 ns unbiased MD simulation of the crystal structure 2ZXE. (**b**) K^+^ bound outward facing model of the pump. During the deocclusion, the extracellular side of the pump opens up and allows water molecules (surface in marine blue) to reach the K^+^ ions (orange spheres) at the ion-binding site. (**c**) The cross-sectional radius profile of the water path leading to the binding site during the occlusion/deocclusion process. The vertical axis marks the position along the *z* axis (membrane normal) in the membrane-pump system. The membrane is centred at *z*=0. The horizontal axis on the bottom is the radius of the solvent accessible cross-sectional area within the pump along z. The radius profile of the occluded state crystal structure in **a** is shown in red. As the extracellular portion of the pump opens, the radius increases at the extracellular side. The radius profile in the final open model is in magenta and those of two intermediate states are shown in green and blue. The *z* positions of the two K^+^-binding sites are indicated by thin black lines. The headgroup (orange) and lipid acyl chain (grey) electron density as a function of *z* (that is, *ρ*(*z*) on the top horizontal axis) is also indicated in **c** with filled curves. The lipid headgroup peak density *z* locations are shown with grey horizontal bars on top of the protein structures in **a**,**b**. (**d**) The outward facing model structure in **b** is taken and split in two parts, M1–4 (left) and M5–10 (right). Each of the two parts is rotated 90° along the shown *z* axis (black dashed axis) so that the bound K^+^ ions are visible. Helices M4 and M6 line the aqueous pathway leading from the extracellular matrix to the binding site and therefore are shown in cartoon representation. Residues that are in direct contact with water molecules are shown in red sticks. Other helices are shown in surface representation.

To help interpret these results on extracellular K^+^ ion binding at the atomic level, we turn to molecular dynamics (MD) simulations. The fraction of membrane potential sensed by the binding of extracellular K^+^ ions can be calculated from Δ〈*Q*_D_〉, the difference in the time-averaged displacement charge of the ion-free and ion-bound outward facing states (see Methods for computational details)^28^. In the absence of a crystal structure of the outward facing Na^+^/K^+^ pump, a model including the entire α-subunit and the transmembrane (TM) segments of the β- and γ- subunits was generated using the crystal structures of the Ca^2+^ SERCA pump as templates (Methods). In the model structure, the two ion-binding sites are accessible to the extracellular solution via a wide aqueous channel (Fig. 5b). Site II is directly exposed to the extracellular solution, while site I is located at the bottom of a deep binding cleft with coordination provided by acidic side chains (Fig. 5d). The binding of the first K^+^ ion in the experiment may reflect occupancy of the two sites in rapid exchange. Assuming it is equally likely that the first K^+^ binds to site I or II, the calculated *λ*_1_ and *λ*_2_ are 0.49±0.12 and 0.37±0.20, respectively. These values are also consistent with estimates based on a linear response approximation (equation (12)), which allows one to visualize the spatial dependence of the applied membrane potential (Methods). Remarkably, the calculations are close to the experimentally determined values (0.46 and 0.27; Fig. 4b and Supplementary Table 1). Further support for the structural model is provided by comparing with a recent crystal structure of the Na^+^/K^+^ pump E_2_ state partially open to the extracellular side with bound ouabain (PDBID: 4HYT)^29^. MD simulations of this structure with ouabain and Mg^2+^ removed showed spontaneous rebinding of Na^+^ to the binding site, leading to the suggestion that this crystal structure resembles the outward facing state of the pump^30^. The model and the crystal structure show similarity, especially in the TM region, where the backbone root mean squared deviation (r.m.s.d.) is 2.7 Å (Supplementary Fig. 5). The computational transition pathway, linking the crystal structure 2ZXE^4^ (occluded with bound K^+^; Fig. 5a) to the model of the outward facing P–E_2_ K_2_ state (Fig. 5b), displays the structural rearrangements expected to occur during the occlusion/deocclusion process. Structural changes in the TM domain mostly involve the M1–M4 helices in the α subunit. More specifically, M1–M3 undergoes a piston-like motion and is pulled towards the intracellular matrix. This makes room for the extracellular portion of M4 (residues W317 to N331), which then tilts and opens up an aqueous channel between M4 and M6 for ions reaching the binding site (Fig. 5a,b). The cross-sectional radius of the water-filled path on the occlusion/deocclusion process is shown in Fig. 5c. This path involves residues F323, G326, A330, E334 on M4 and T804, I807, L808 and D811 along M6 (Fig. 5d). Interestingly, several of these residues are known to be along the open conducting ion channel arising in the palytoxin-bound conformation^31^,^32^,^33^.

## Discussion

The goal of this study is to combine functional and computational approaches to understand the mechanism of extracellular K^+^ uptake by the Na^+^/K^+^-ATPase. Functionally, we dissected pump-mediated K^+^-dependent pre-steady-state currents corresponding to external K^+^ binding/unbinding and occlusion/deocclusion. Our steady-state and kinetic experimental data are best described by a model in which both external K^+^ ions bind sequentially^34^,^35^ before they are simultaneously occluded in a single transition with occlusion and deocclusion rates of ∼13,000 s^−1^ and ∼2,000 s^−1^, respectively. As previously observed^23^,^35^,^36^,^37^,^38^,^39^,^40^,^41^, our measurements show that external K^+^ binding by the pump (*K*_d1(0)_=2.6 mM, *K*_d2(0)_=42.7 mM) have orders of magnitude higher apparent affinities than those determined for external Na^+^, supporting forward directionality of the pump transport cycle. Yet, these *K*_d(0)_ values appear to be larger than the estimated extracellular apparent affinity for K^+^ when squid pump turnover rates were monitored in the absence of external Na^+^ by unidirectional Na^+^ efflux (0.5 mM)^23^. This discrepancy likely originates from the cations used to substitute Na^+^. If we use *N*-methyl D-glucamine, as previously^23^, instead of tetramethyl ammonium (TMA), much faster relaxations rates were measured with 1 or 2 mM K^+^ (Supplementary Fig. 6). These results suggest that our *K*_d(0)_ values are possibly overestimated (at least three-fold) by TMA competition for the extracellular access channel. The *λ* sensed by the first and second K^+^ ions are 0.46 and 0.27, respectively. These values are within the range of previous estimations using steady-state Na^+^/K^+^-ATPase current in which single weighed *λ* for K^+^ were reported^16^,^17^,^19^,^22^,^42^,^43^.

The steady state and kinetics of the Na^+^/K^+^-ATPase’s interactions with external Na^+^ and K^+^ appear to be fundamentally different. Our measurements show that external K^+^ binding by the pump has orders of magnitude higher apparent affinities and much faster occlusion rates than Na^+^ occlusion. Our MD simulations show a wide water-filled access channel connecting the binding sites to the external solutions, consistent with the experimental rapid binding rate and the small fraction of electric field sensed by the external K^+^ ions. These differences accelerate forward pumping under the normally adverse ionic conditions (higher [Na^+^]_o_ than [K^+^]_o_), thus keeping the adequate homeostasis of the cell.

## Methods

### Electrophysiology

Humboldt squid (*Dosidicus gigas*) were collected in the coast of Valparaiso, Chile. Giant axon preparation and electrophysiology were performed as in Castillo *et al.*^6^ In brief, giant axons (950–1,200 μm diameter) were mounted in a chamber that contains two guard pools and a central test pool. A dialysis system containing two 100-μm platinized platinum wires is inserted along the axon. Membrane voltage is measured and controlled between an internal glass electrode filled with 0.6 M KCl solution and an external reference electrode filled with a 3-M KCl/agar. The current is collected in the central chamber. In the present work, giant axons were voltage clamped, internally dialysed and externally superfused at 20 °C with solutions designed to restrict the pump to K^+^/K^+^ exchange conditions. The intracellular solution was (in mM; pH adjusted with HEPES): 100 K-HEPES, 25 K-PO_4_, 25 *N*-methyl-D-glucamine (NMG)-HEPES, 50 glycine, 50 phenylpropyltriethylammonium-sulphate, 5 dithiothreitol, 2.5 1,2-bis(2-aminophenoxy)ethane-*N*,*N*,*N*9,*N*9-tetraacetic acid (BAPTA), 25 Mg-HEPES and 5 Mg-ATP. To record robust signals, 5 mM ATP and 25 mM Pi were required. ATP (5 mM) may influence the E_2_(K_2_) → E_2_(K_2_). ATP reaction, but as this rate has only been estimated indirectly, its effect on the time course of the K^+^-mediated charge movement is difficult to assess. If there is any influence, the relaxation rates of our measured K^+^-transient currents would be faster than in the absence of ATP. The extracellular solution was (in mM): 1 to 8 KCl, 400 tetramethylammonium-chloride (TMA-Cl) or NMG-Cl, 75 CaCl_2_, 1 3,4-diaminopyridine, 2 × 10^−4^ tetrodotoxin, 5 Tris-HEPES and 0.05 EDTA (pH 7.7). Osmolalities of all solutions were ∼950 mOsmol kg^−1^. Voltage pulses were generated and currents recorded using a 14-bit A-D/D-A converter board (A4D1, Innovative Integration) with in-house developed software. Currents were filtered at 80–150 kHz, then sampled at 500 kHz to 2 MHz. Current records were acquired after online analogue capacitive transient subtraction. The pump-mediated currents were isolated from the total membrane current by using H_2_DTG, which specifically and reversibly block squid Na^+^/K^+^ pumps^23^. H_2_DTG-sensitive currents were analysed with in-house software. Global fits were carried out with programmes written in Matlab (Supplementary Data 1).

### Kinetic modelling

The probabilities of occupancies for each state of the model shown in Fig. 4d are denoted as *S*_1_=P−E_2_, *S*_2_=P−E_2_·K_1_, *S*_3_=P−E_2_·K_2_ and *S*_4_=E_2_(K_2_), the equations to solve are:




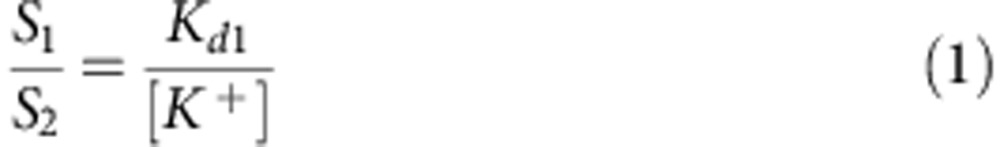







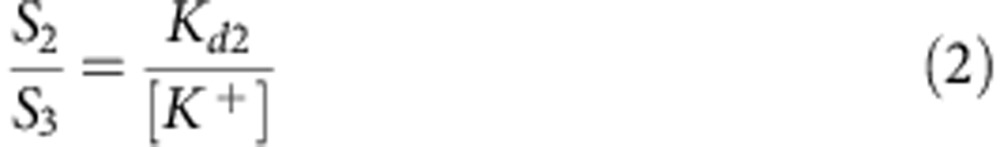







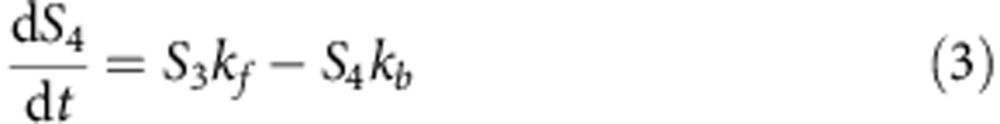




and




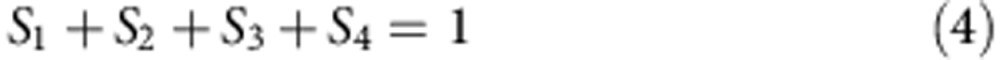




Where the voltage dependence of the dissociation constants are given by:




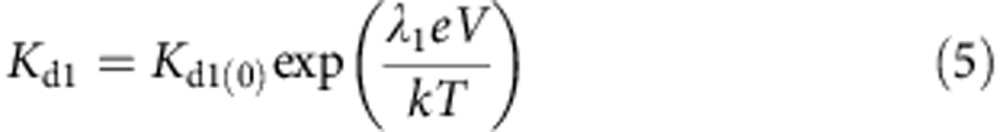




and




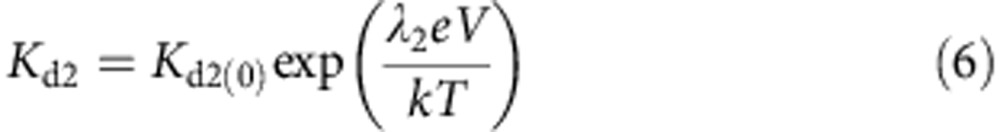




The solution for the charge *Q* is given by:




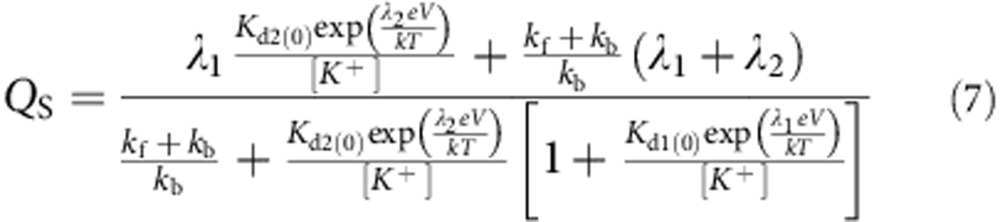




and the rates are given by:




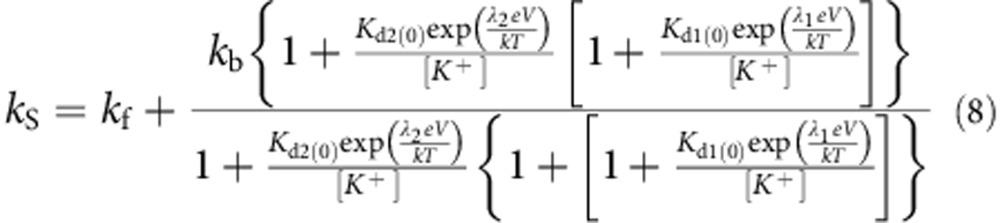




### Modelling of the outward facing Na^+^/K^+^ pump structure

The Ca^2+^ SERCA pump X-ray structures were used as templates to guide the modelling of an outward facing Na^+^/K^+^ pump structure (P−E_2_ K_2_ in Fig. 1). Because they both are P-type ATPases sharing 39% sequence similarity, each state along the cycle of the Na^+^/K^+^ pump is expected to have its counterpart along that of SERCA. However, which structures of SERCA correspond to the two available Na^+^/K^+^ pump x-ray structures, E_1_ (PDBID 3WGV)^1^ and E_2_ (PDBID 2ZXE)^4^ must be determined. For this purpose, structural alignments of the TM domains were carried out for 3WGV and 2ZXE with all the SERCA pump structures and this was done using TM-align^44^. The corresponding SERCA pump structure was chosen as the one displaying the lowest r.m.s.d. for the protein backbone in the TM region. For the E_1_ state structure of the Na^+^/K^+^ pump, the corresponding SERCA structure is E_1_.Ca_2_ (PDBID 1SU4)^45^, with a backbone r.m.s.d. between the two of 2.47 Å. For the Na^+^/K^+^ pump E_2_(K_2_) state, the corresponding SERCA structure is E_2_.P with a backbone r.m.s.d. of 2.89 Å (PDBID: 1WPG)^46^. With two states in the Na^+^/K^+^ pump cycle unambiguously assigned onto those of the SERCA pumping cycle, the correspondence for the remaining states could be determined. The corresponding SERCA pump state for the desired Na^+^/K^+^ pump P−E_2_ K_2_ structure is E_2_−P (PDBID 3B9B)^47^. Close inspection of this X-ray structure of SERCA reveals that the ion-binding site is indeed open to the sarcoplasmic reticulum lumen, which corresponds to the extracellular matrix in the case of a Na^+^/K^+^ pump.

A conformational transition pathway linking the two SERCA pump structures (E_2_.P 1WPG and E_2_-P 3B9B) corresponding to the E_2_(K_2_) and P−E_2_ K_2_ states of the Na^+^/K^+^ pump was generated using the ANMPathway online server^48^ (http://anmpathway.lcrc.anl.gov/anmpathway.cgi). This method produces a coarse-grained transition pathway of the Cα backbone by assuming mixing of two simplified anisotropic network models for each of the stable end states. The curvilinear pathway, discretized into 58 image snapshots distributed at equal r.m.s.d. interval, was then used to construct an all-atom model of the system using targeted MD (TMD) simulations.

To obtain an all-atom model of the outward facing P−E_2_ K_2_ state structure, we performed a series of TMD simulations. An initial system containing the E_2_(K_2_) X-ray structure (PDBID 2ZXE) and POPC membrane was assembled with the CHARMM-GUI membrane builder module^49^,^50^,^51^ with 0.15 M KCl and 0.15 M NaCl in the solution. On the basis of pKa calculations with PROPKA web server^52^,^53^,^54^,^55^ and the previous selectivity studies^56^, residues E334, E786, D815, D933, E960 and E961 were protonated. The complete system was equilibrated for 675 ps while reducing constraints on heavy atom positions followed by a 300-ns production run without any constraints. The TMD simulations comprised 58 segments along the complete curvilinear pathway, one following another. Each segment was 1 ns long during which the system was steered from an initial element *i* to a target element *i*+1. The steering forces of the TMD were only exerted on the Cα atoms in the α-subunit TM region corresponding to those in the template SERCA structure, allowing the side chains and the other subunits (β and γ) to freely relax along the pathway. The ectodomain of the β-subunit was not included to reduce the computational cost. This procedure has the advantage of keeping the side chains of the outward facing P−E_2_ K_2_ state model physically consistent with those of the E_2_(K_2_) X-ray structure (PDBID 2ZXE) by continuity of the pathway.

The initial relaxation of 675 ps was performed using the NAMD2.9 simulation package^57^ with the input scripts from the CHARMM-GUI membrane builder module^49^,^50^,^51^, followed by an extensive 300-ns equilibration with no restraints conducted using the special-purpose supercomputer Anton^58^. The volume of the periodic cell was kept constant and the temperature was set to 303.15 K using the Nosé–Hoover thermostat^59^. The lengths of all bonds involving hydrogen atoms were constrained using M-SHAKE^60^. The cutoff of the van der Waals and short-range electrostatic interactions was set to 15.31 Å. Long-range electrostatic interactions were evaluated using the *k*-space Gaussian split Ewald method^61^ with a 64 × 64 × 64 mesh. The integration time step was 2 fs. The r-RESPA integration method^62^ was employed and long-range electrostatics were evaluated every 6 fs. A TM voltage of −90 mV was applied in the simulation to simulate the physiological condition under which the pump functions.

All the TMD simulations were performed at constant volume and temperature using the NAMD2.9 simulation package^57^. The PARAM27 all-atom force field of CHARMM^63^ with a modified version of dihedral cross-term correction^64^ was used for the protein and the C36 lipid force field^65^ was used for POPC. Water molecules were modelled with the TIP3P potential^66^. The simulation temperature was set to 303.15 K using Langevin Dynamics with a damping coefficient of 1 ps^−1^. The van der Waals interactions were smoothly switched off at 10–12 Å by a force switching function^67^ and the electrostatic interactions were calculated using the particle-mesh Ewald method with a mesh size of ∼1 Å. A TM voltage of −90 mV was applied. The force constant on each Cα atom during the TMD simulations was set to 50 kcal mol^−1^ Å^−2^.

### Calculation of fractional membrane potential of K^+^ binding

The fraction of membrane potential sensed by K^+^ at the binding site (*λ*_1_ and *λ*_2_) was calculated from MD simulations based on atomic models of the pump in a membrane with explicit solvent using the Q-route described previously^28^. The Q-route provides one way to compute the gating charge and *λ* on extracellular K^+^ binding. In the Na^+^/K^+^ pump, the gating charge (Δ〈*Q*_D_〉) of each ion binding directly reflects the *λ* this ion feels going from the bulk solution to a given binding site. For an atomic model, the instantaneous displacement charge, *Q*_D_(*t*), for a given configuration of the system is expressed as the sum of all the atomic charges and their positions along the direction of the applied electric field, the *z* axis in this case, normalized by the simulation cell dimension *L*_*z*_ along the *z* axis (assumed to be perpendicular to the bilayer membrane),




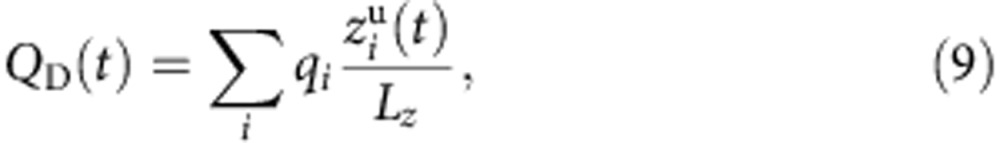




where *q*_*i*_ and 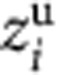 are the atomic charge and ‘unwrapped’ *z* coordinate of the *i*th atom, respectively. By virtue of the applied periodic boundary conditions, the coordinates of an ion exiting the simulation box from the top (bottom) are wrapped around to re-enter the box from the bottom (top) in the output trajectory produced by Anton. However, unwrapped *z* coordinates must be used to correctly calculate the displacement charge *Q*_D_ (ref. 28). In practice, because there is no ion flux through the membrane, the issue of unwrapping coordinates can be addressed simply by shifting all ions in the simulation system above (or below) a chosen position along the z axis so that all the ions appear in the extracellular (or intracellular) side relative to the membrane bilayer. These unwrapped *z* coordinates of the ions (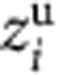) can then be used for the *Q*_D_ calculations with equation (9). The displacement charge (〈*Q*_D_〉) for a given simulation trajectory is the time average of *Q*_D_(*t*) calculated from all the snapshots. Four simulation systems were set up to compute the changes in displacement charge, Δ〈*Q*_D_〉, on extracellular K^+^ binding. System MD_11 was taken from the last snapshot of the TMD simulations with the K^+^ bound and outwardly faced Na^+^/K^+^ pump structure embedded in a POPC bilayer. To generate the second system, MD_10, the K^+^ ion at site II was taken out of the binding site and randomly placed in the bulk solution. The third system, MD_01, was generated in a similar way with the K^+^ ion at site I taken out. In the fourth system (MD_00), both of the bound K^+^ ions were removed from the binding site and placed randomly in the bulk solution. Each of these four systems was simulated for 120 ns on Anton^58^ with the same simulation protocol described above and additional positional constraints on the backbone atoms in the protein. The gating charge on ion binding was calculated as the difference in 〈*Q*_D_〉 between two systems. It is worth noting that since the binding site is solvent accessible in the outward facing model structure, occasionally cations other than the bound K^+^ can enter the access channel. If these snapshots were included in the displacement charge calculation, it would skew the final result. Therefore, trajectory snapshots with such a condition were excluded when calculating 〈*Q*_D_〉. The first 20 ns of trajectory snapshots were also not included to eliminate the impacts from fluctuations due to the initial relaxation of the system. The calculated 10-ns block averages of 〈*Q*_D_〉 in systems MD_00, MD_10, MD_01 and MD_11 are −6.67±0.08, −7.26±0.13, −7.06±0.14 and −7.53±0.18, respectively. The Δ〈*Q*_D_〉 for the first K^+^ binding at site I is 0.59 and the value becomes 0.39 if site II is the first ion-binding site. Assuming it is equally likely for the first K^+^ to bind at either site, then the Δ〈*Q*_D_〉 for the first ion binding becomes




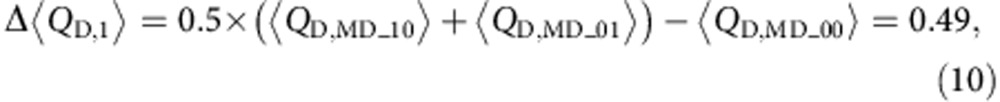




and the Δ〈*Q*_D_〉 for the second K^+^ binding is




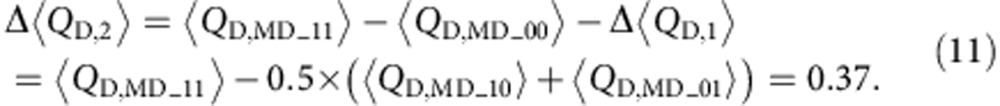




The s.e. is given in Supplementary Table 1. Although this is an assumption, a study is already underway to determine the K^+^ binding affinity at sites I and II.

An alternative route based on a linear response approximation is to calculate directly the component of the mean electrostatic potential associated with the applied membrane potential^28^. Starting from the last snapshot of the TMD simulations, the two bound K^+^ ions were removed from the binding site and placed randomly in the bulk solution. This system served as a starting point for two simulations with different TM potentials at −90 and 90 mV respectively. The simulations were carried out with NAMD2.9 (ref. 57) and harmonic constraints with a spring constant of 1 kcal mol^−1^ Å^−2^ were applied on the backbone atoms in the protein to prevent large conformational change. Constraint forces were also applied to repel mobile ions from entering the access channel, which would bias the final results of the electrostatic potential calculation. Other simulation details followed the NAMD simulation protocol mentioned in the previous section. The duration of each simulation was 24 ns and snapshots were taken every 2 ps for the electrostatic potential calculations. The VMD plugin, PMEpot^68^, was used to compute the total electrostatic potential Φ(**r**) in the system. The dimensionless fraction of membrane potential at position **r**, *ϕ*_mp_(**r**), was calculated from the difference,




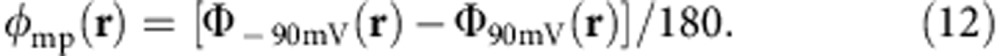




The contributions to the potential arising from the interface in the system cancel out in the subtraction and *ϕ*_mp_ (**r**) is obtained after normalizing the difference in Φ(**r**) by 180 mV. The drop in membrane potential fraction at the K^+^-binding sites was given by the difference between *ϕ*_mp_ in the bulk solution at the extracellular side and in the binding site. While it relies on a linear response approximation, the calculated *ϕ*_mp_ provides a nice simplification to help visualize the underlying features of the systems. The *ϕ*_mp_ (*x*, *z*) map of a cross section of the system at *Y*=−44.7 Å in between the two K^+^-binding sites is shown in Supplementary Fig. 7. It clearly shows the *ϕ*_mp_ drop from the bulk solution to the binding sites. The trajectories are divided into 4-ns chunks and the *ϕ*_mp_ difference is calculated for each trajectory block. The final averaged *ϕ*_mp_ differences along with the s.e. are tabulated in Supplementary Table I.

## Additional information

**How to cite this article**: Castillo, J. P. *et al.* Mechanism of potassium ion uptake by the Na^+^/K^+^-ATPase. *Nat. Commun.* 6:7622 doi: 10.1038/ncomms8622 (2015).

## Supplementary Material

Supplementary Information Supplementary Figures 1-7, Supplementary Table 1 and Supplementary References

Supplementary Data 1 Scripts for data analysis
