# Supplementary material for: Mechanism of potassium ion uptake by the Na+/K+-ATPase
Source: Nat Commun. 2015 Jul 24;6:7622. doi: 10.1038/ncomms8622 (PMC4515779; doi:10.1038/ncomms8622)
Supplement: Supplementary Information — Supplementary Figures 1-7, Supplementary Table 1 and Supplementary References [file ncomms8622-s1.pdf]

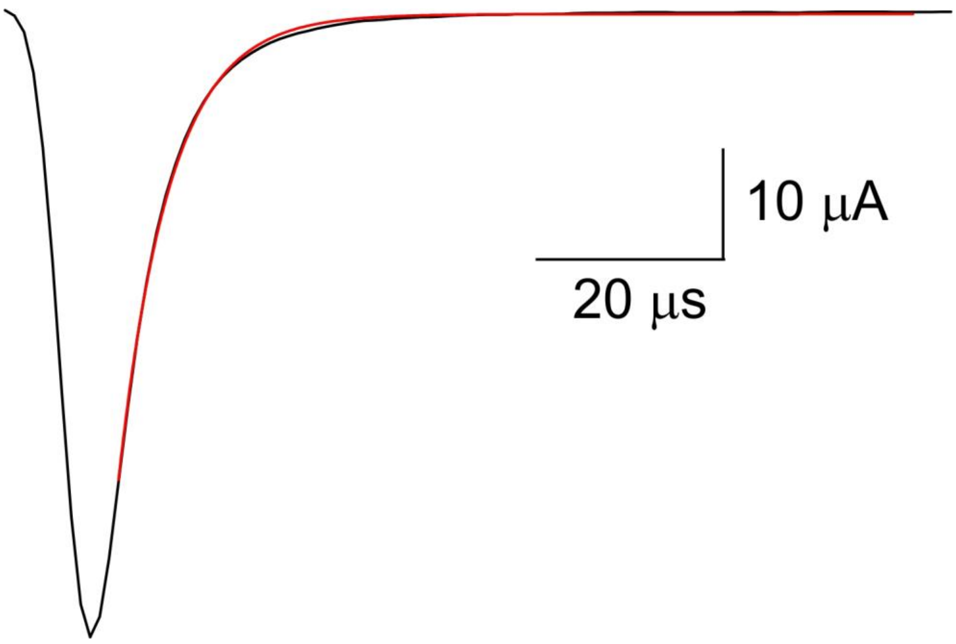

Supplementary Figure 1. Voltage clamp speed. Capacity membrane current in response to a 4-mV voltage step (black). Solid red line corresponds to a mono-exponential fit with a time constant of 5.5 µs. Signal was sampled at 1 MHz and filtered at 200 kHz.

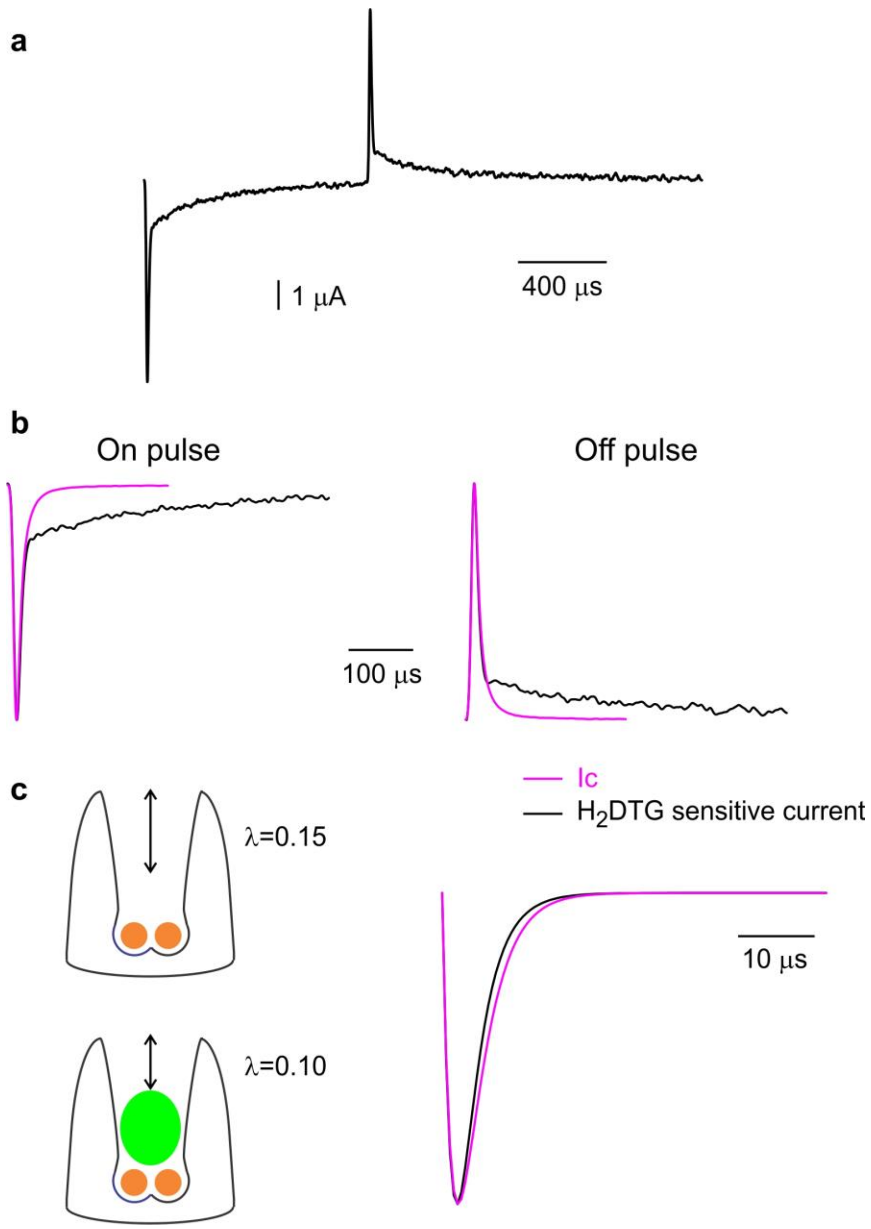

Supplementary Figure 2. Fast component of K^+ translocation. a, H_2DTG sensitive transient current in response to a 1-ms voltage step to -160 mV and back to 0, in the presence of 1 mM external K^+. The fast component has similar time course and magnitude at the On and Off. **b,** Superposition of the H_2DTG-sensitive transient current (black) with the scaled (6 times smaller) capacity transient (magenta; 4-mV step) from the same axon at the On and Off voltage steps. The time courses of the fast component of K^+ translocation is indistinguishably from the voltage clamp speed. **c,** Model: H_2DTG binding leaves an open access channel with a smaller electrical depth than in its absence (left). On the right is shown a simulation of this model. Black solid line represents a H_2DTG-sensitive transient current from two simulations carried out with 1000 pumps/µm^2, electrical depths as shown in left and a voltage clamp speed of 3 µs. The voltage step was from 0 mV to -200 mV. Magenta solid line represents a scaled (510 times smaller) capacity transient for the same voltage step and clamp speed, and assuming a 1 µF/cm^2.

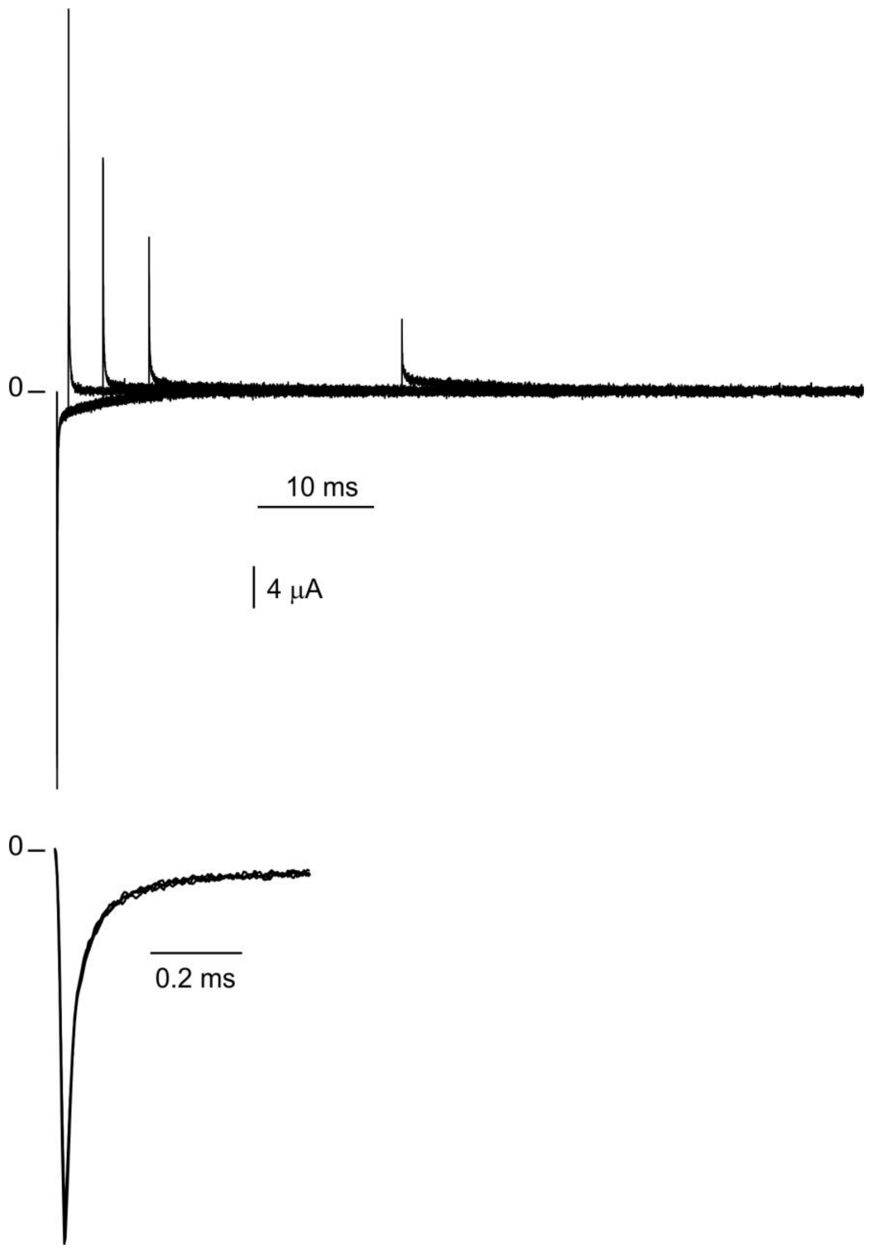

Supplementary Figure 3. Fast and slow components of Na^+ translocation are kinetically dependent. Superimposed H_2DTG sensitive transient currents in response to voltage jumps from a holding potential of 0 to -120 mV with step durations of 1, 4, 8 and 30 ms and returning back to 0 mV. At the steps' onset, all four transient currents have similar amplitudes of the fast spike, as shown at bottom in an expanded time scale; which indicates that the population of pumps was at similar initial conditions prior to the voltage jump to -120 mV. Upon return top 0 mV, the magnitude of the fast component decreased as the step duration increased, a landmark of distinct and sequential occlusion steps for Na^+. Methods: This experiment was performed with a *Dosidicus gigas* axon using ionic conditions that restrict Na^+/K^+ pumps to states associated with binding/release and occlusion/deocclusion of external Na^+[(1,2)]. External [Na^+] was 100 mM, sampled at 400 kHz and filtered at 80 kHz.

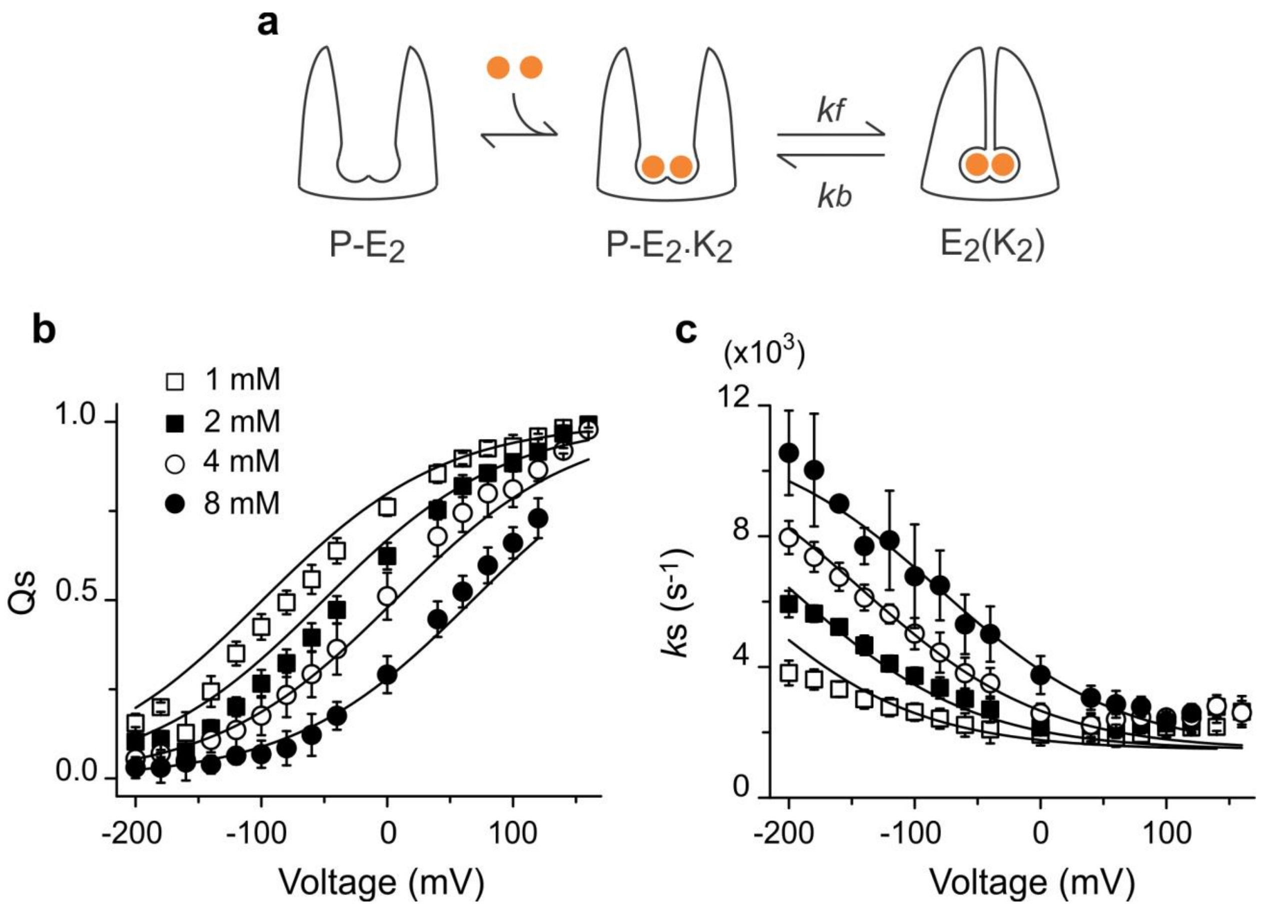

Supplementary Figure 4. Single binding step model. a, Cartoon model representing two K^+ binding and occluding simultaneously. Charge quantities (**b**) and relaxation rates (**c**) data were fitted to this model (solid lines). Best fit parameter values were: K_d = 19.2 mM, λ = 0.25, k_f = 9780 s^{-1}, k_b = 1460 s^{-1} and n = 1.36 (r^2 =0.96; cf. Fig. 3b). n=9, 12, 8 and 5 for 1, 2, 4 and 8 mM K^+, respectively; bars represent SD (when not shown, SD was smaller than the symbol size).

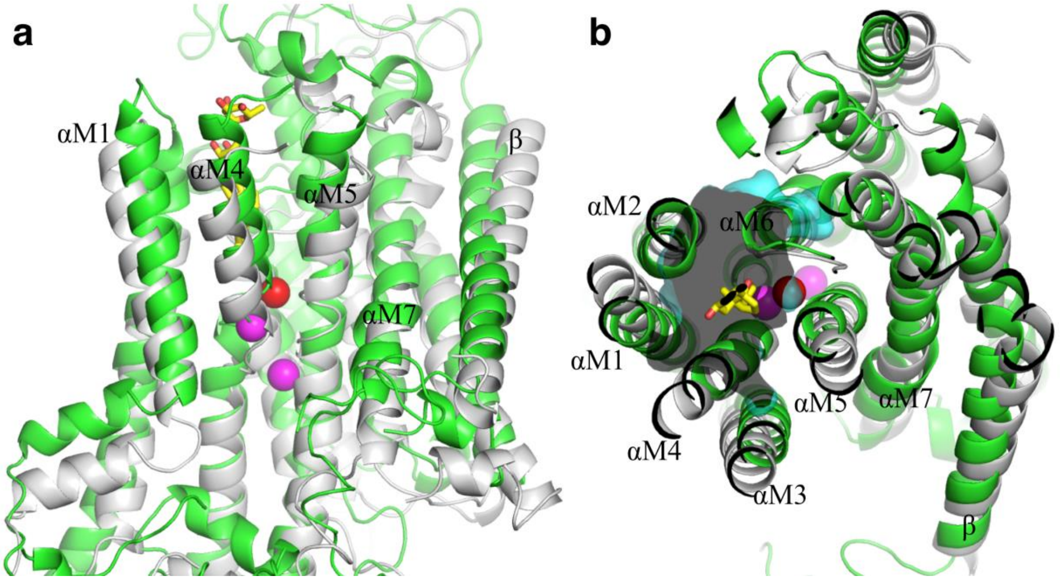

Supplementary Figure 5. Overlay of the outward facing Na^+/K^+ pump model (*white*) and the ouabain bound E2 state crystal structure (*green*)[3] viewed from **a**, the side and **b**, the extracellular side. The ions including bound K^+ (*magenta*) and Mg^{2+} (*red*) are shown in sphere presentation. The ouabain molecule is shown in stick presentation (*yellow*). Water molecules accessing the binding site in the model are shown in surface presentation (*cyan*).

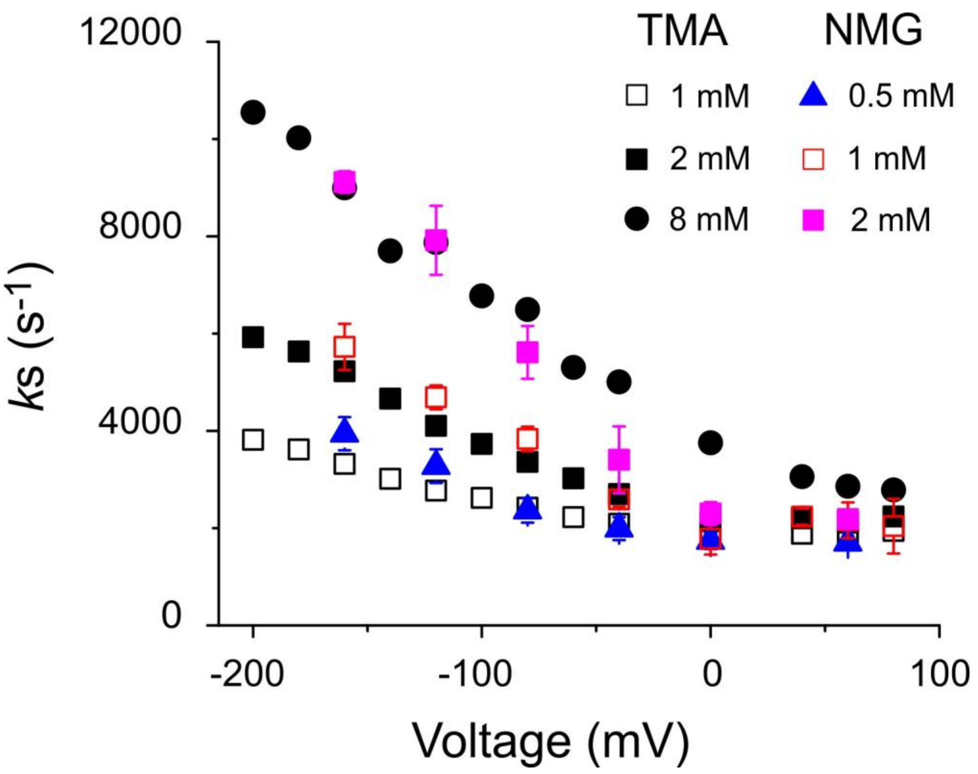

Supplementary Figure 6. Influence of external monovalent cation substitute on the K^+ translocation's relaxation rates. At negative potentials and comparable external K+ concentrations, the relaxation rates are substantially faster when N-methyl-D-glucamine (NMG) substituted 400 mM Na^+ than those measured using tetramethyl ammonium (TMA) instead. These results suggest that TMA is competing with K^+ for accessing the extracellular access channel of the pump. We were not able to perform experiments with higher K^+ in NMG solutions because rates become comparable to the clamp speed. n=4, 4 and 3 for 0.5, 1 and 2 mM K^+, respectively; bars represent SD (when not shown, SD was smaller than the symbol size).

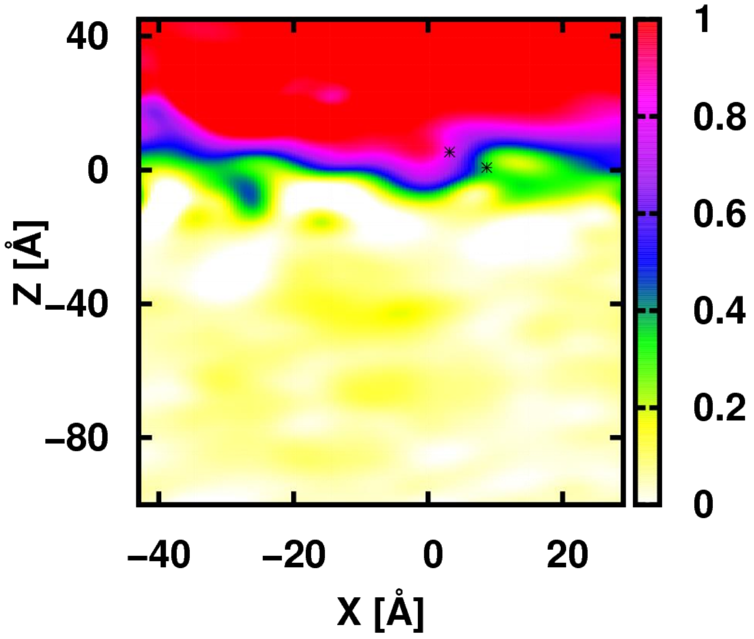

Supplementary Figure 7. The electrostatic potential fraction (ϕ_{mp}) map of the cross-section of the system along the *X*- and *Z*-directions at *Y*=-44.7 Å. This is between the *Y*-positions of the two K^+ binding sites (Y_I = -44.0 Å and Y_{II} = -45.4 Å). ϕ_{mp} is calculated based on Eq 4 in the Methods section. The *X*- and *Z*-position of the two binding sites are shown as black stars on the map. The membrane center is at *Z* = 0. The extracellular side faces the positive *Z*-direction.

Supplementary Table I. The membrane potential fraction change (λ) upon extracellular K^+ binding from experiments and calculations.

	**1st K^+**	**2nd K^+**
Experimental fit: value (95% Confidence)	0.46 (0.43, 0.48)	0.27 (0.25, 0.29)
ΔQ_D (average ± SE)	0.49 ± 0.12	0.37 ± 0.20
Linear response (average ± SE)	0.58 ± 0.17	0.18 ± 0.11

References

1 Gadsby, D. C., Bezanilla, F., Rakowski, R. F., De Weer, P. & Holmgren, M. The dynamic relationships between the three events that release individual Na^+ ions from the Na^+/K^+-ATPase. *Nat. Commun.* **3**, 669 (2012).

2 Castillo, J. P. *et al.* Energy landscape of the reactions governing the Na^+ deeply occluded state of the Na^+/K^+-ATPase in the giant axon of the Humboldt squid. *Proc. Natl. Acad. Sci. U S A* **108**, 20556-20561 (2011).

3 Laursen, M., Yatime, L., Nissen, P. & Fedosova, N. U. Crystal structure of the high-affinity Na^+K^+-ATPase-ouabain complex with Mg^{2+} bound in the cation binding site. *Proc. Natl. Acad. Sc.i U S A* **110**, 10958-10963 (2013).
